# Hepatitis E virus infection during pregnancy

**DOI:** 10.1186/s12985-020-01343-9

**Published:** 2020-06-10

**Authors:** Chunchen Wu, Xiaoxue Wu, Jianbo Xia

**Affiliations:** Department of Laboratory Medicine, Maternal and Child Health Hospital of Hubei Province, Tongji Medical College, Huazhong University of Science and Technology, Wuhan, 430070 People’s Republic of China

**Keywords:** Hepatitis E virus (HEV), Hepatitis E, Pregnancy

## Abstract

**Background:**

Hepatitis E virus (HEV) generally causes self-limiting viral hepatitis. However, in pregnant women, HEV infection can be severe and has been associated with up to 30% mortality in the third trimester. Additionally, HEV infection in pregnancy is also associated with high rates of preterm labor and vertical transmission.

**Main body:**

HEV is now recognized as a global health problem in both developing and industrialized countries. HEV can be transmitted via the fecal-oral route, zoonotic route, and blood transfusion route. An altered immune status, hormonal levels, and viral factors may be related to the severity of the disease. Currently, no established treatment is available for HEV in pregnant women. A Chinese vaccine has been demonstrated to be protective against HEV in the general population and seems to be safe in pregnancy; however, its safety and efficacy in a large population of pregnant women remain to be determined.

**Conclusion:**

This review summarizes the current knowledge about HEV infection during pregnancy and focuses on the epidemiology, clinical manifestations, mechanisms underlying severe liver injury, and management and prevention of HEV infection during pregnancy. Considering that HEV infection during pregnancy may result in poor outcomes, screening for and monitoring HEV infection early in pregnancy should be taken into account. In addition, a better understanding of the pathogenesis will help to develop potential treatment strategies targeting HEV infection in pregnancy.

## Background

Hepatitis E virus (HEV) is a hepatotropic infectious agent that generally causes self-limiting acute hepatitis in healthy adults and chronic hepatitis in immunocompromised individuals [1]. Annually, there are an estimated 20 million HEV infections, 3.3 million symptomatic hepatitis E cases, and 60,000 deaths worldwide [1, 2]. In addition to hepatic manifestations, extrahepatic manifestations, including pancreatitis, neurological symptoms, hematological disorders, glomerulonephritis, and mixed cryoglobulinemia have been associated with HEV infection [1, 3]. HEV is classified into 8 major genotypes, with genotypes 1–4 being the predominant strains involved in human infections. Genotypes 1 (HEV-1) and 2 (HEV-2) infect only humans and are transmitted via the fecal-oral route [1, 4], whereas genotypes 3 (HEV-3) and 4 (HEV-4) are zoonotic and are contracted through the consumption of undercooked pork and wild boar meat [5]. Although swine are the most commonly implicated source of HEV-3 and HEV-4, other animals including shellfish, deer, and rabbits can serve as a source of infection [4, 6–9]. Therefore, HEV-1 and HEV-2 are often associated with epidemics in developing countries due to poor hygiene and sanitation. In contrast, HEV-3 and HEV-4 are prevalent in industrialized countries and are associated with sporadic and clustered cases of hepatitis E in these regions [1, 4]. In recent years, the transmission of HEV-3 and HEV-4 through blood transfusions has been increasingly reported in Europe [1, 4, 10], prompting many countries to consider the screening of blood products for HEV [11]. For these reasons, HEV is now recognized as a global health problem in both developing and industrialized countries [1, 12]. Current therapeutics used to treat HEV infection include the nucleoside analog ribavirin and interferon-α (IFN-α). Ribavirin therapy can be fairly efficient in most cases of chronic hepatitis E [1, 2, 13, 14]. In some specific situations, interferon has also been used successfully [2]. However, until now, there have been no specific direct-acting antiviral agents against HEV, particularly for pregnant women [1, 15]. A recombinant hepatitis E vaccine, HEV 239, has been demonstrated to be well tolerated and effective in the prevention of hepatitis E in China; however, it is currently only approved for use in China and is not yet available elsewhere [2, 16, 17].

HEV infection can cause fulminant hepatitis failure (FHF), especially in pregnant women, with a mortality rate of up to 30% [1, 18–20]. In addition, HEV can be vertically transmitted from infected mothers to their infants, with significant perinatal morbidity and mortality [20–22]. Ribavirin and IFN-α are contraindicated in pregnancy because of the risk of teratogenicity [23–25]. Therefore, only supportive care is provided for pregnant women with HEV infection. In this review, we provide a summary of the current knowledge regarding HEV and highlight HEV infection during pregnancy, which has been poorly understood until now and deserves the utmost attention.

## Main text

### Molecular virology of HEV

**Fig. 1:**
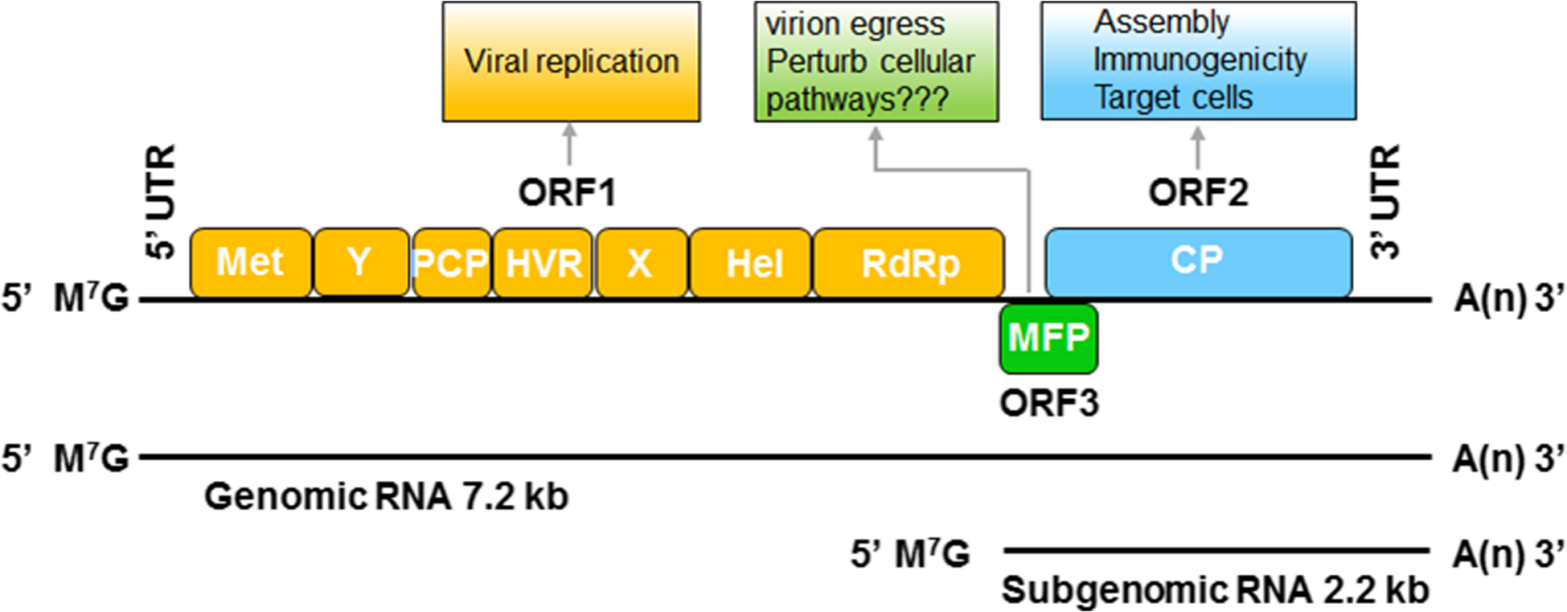
Schematic description of the hepatitis E virus (HEV) genome and viral proteins. The HEV genome is a single-stranded, positive-sense, linear RNA that is approximately 7.2 kb in length. The RNA genome consists of a 5′ untranslated region (UTR), three open reading frames (ORFs) and a 3′ UTR. ORF1 comprises approximately 70% of the genome (5109 bp) and encodes nonstructural polyproteins, such as methyltransferase (Met), Y-domain (Y), PCP, hypervariable region (HVR), macrodomain (X), RNA helicase (Hel) and RNA-dependent RNA polymerase (RdRp), which are necessary for replication. ORF2 and ORF3 partially overlap and are translated from a single subgenomic RNA that is approximately 2.2 kb in length. ORF2 encodes a viral capsid protein that is required for viral entry, assembly and immunogenicity. ORF3 encodes a small multifunctional phosphoprotein (MFP) that is required for virus egress from cells and proposed to perturb numerous cellular pathways

HEV belongs to the family of *Hepeviridae* and is the sole member of the genus *Orthohepevirus*. HEV is icosahedral in shape and can exist in both nonenveloped and enveloped (eHEV) forms, with diameters of approximately 30 nm and 40 nm, respectively [1, 12]. These two forms of HEV particles may be transmitted via different routes and possess distinct characteristics in the viral life cycle [1, 12]. The HEV genome is a single-stranded, positive-sense, linear RNA that is approximately 7.2 kb in length [26]. The RNA genome consists of a 5′ untranslated region (UTR), three open reading frames (ORFs) and a 3′ UTR. ORF1 comprises approximately 70% of the genome (5109 bp) and encodes nonstructural polyproteins, such as methyltransferase (Met), Y-domain (Y), papain-like cysteine protease (PCP) [27], hypervariable region (HVR) [28], macrodomain (X), RNA helicase (Hel) [29, 30] and RNA-dependent RNA polymerase (RdRp) [31, 32], those are necessary for replication [1, 17, 33]. ORF2 and ORF3 partially overlap and are translated from a single subgenomic RNA [34]. ORF2 encodes both glycosylated ORF2 antigen and viral capsid protein [35, 36]. ORF3 encodes a small multifunctional phosphoprotein that is required for virus egress from cells and proposed to perturb numerous cellular pathways [12, 17] (Fig. 1).

The study of HEV replication has been limited to efficient cell culture systems, and replication within the host is not yet fully understood. The HEV capsid protein is believed to be essential for binding to an unknown cellular receptor to initiate viral entry [37]. The eHEV may have a distinct mechanism of viral entry, considering that it is enveloped and capsid protein is not exposed on the surface [38]. After entering the target cell, the virus is uncoated, and the viral RNA genome is released into the cytoplasm. Following this, viral nonstructural proteins are translated from ORF1 [39]. A negative-sense intermediate RNA is synthesized based on the positive-sense viral RNA genome with the help of RdRp; this product, in turn, serves as a template to produce 7.2-kb positive-sense progeny viral RNA as well as 2.2-kb subgenomic RNA [31, 40]. Subsequently, the subgenomic RNA acts as a template to translate capsid proteins and phosphoproteins [37]. The genomic RNA is contained within the capsid proteins, and new virions are generated and finally released by mechanisms still unclear [17, 41].

### Epidemiology of HEV infection in pregnancy

HEV infection is associated with high incidence and mortality (principally due to fulminant hepatitis) in pregnant women according to the majority of clinical studies and case reports from developing countries [1, 4, 10, 42]. A prospective field study carried out by Khuroo et al. showed that hepatitis E developed in 36 (17.3%) of 208 pregnant women, as compared to 71 (2.1%) of 3350 nonpregnant women and 107 (2.8%) of 3822 men. FHF developed in 8 (22.2%) of the 36 pregnant women with hepatitis E. In contrast, none of the nonpregnant women showed the occurrence of fulminant hepatic failure [43]. For the first time, this study revealed that the incidence of hepatitis E and fulminant rates were higher in pregnant women than nonpregnant women and men. In another study, HEV infection was observed in 57.5 and 46% of pregnant and nonpregnant women, respectively. Moreover, 58% of the HEV-infected pregnant women developed FHF. The mortality rate was highest (56%) among HEV-infected FHF cases during the third trimester of pregnancy [44]. This study indicated that pregnant women, especially those in the second and third trimesters, are more commonly affected during epidemics than the general population [42]. Various studies from different regions of the Indian subcontinent reported that the prevalence rate of HEV in acute viral hepatitis during pregnancy ranged from 58 to 86% [44–46]. HEV infection can result in up to 30% mortality among pregnant women in their third trimester [1, 18–20]. HEV-1 and HEV-2 are likely responsible for most of the HEV outbreaks because they are the most prevalent genotypes in developing countries [10, 42]. Additionally, HEV infection in pregnancy is also associated with high rates of preterm labor and vertical transmission [10, 47, 48]. The frequent occurrence of other complications, such as disseminated intravascular coagulation (DIC), is also associated with HEV infection in pregnant women [4]. For industrialized countries, there are few reported cases in pregnant women [49–51]; HEV-3 and HEV-4 are associated with these cases [50, 51]. Unlike HEV-1 and HEV-2, HEV-3 and HEV-4 do not appear to cause fatal infections with fulminant hepatitis in pregnant women [10, 49]. However, considering that the limitation of these studies was the small number of cases, other studies are needed to clarify this result.

China is an epidemic area for HEV, and HEV-4 is currently the dominant cause of hepatitis E [52, 53]. HEV infection has considerable potential for transmission in pregnant women in China. It was estimated that the positivity rates of anti-HEV IgM and anti-HEV IgG were 2.6 and 16.2%, respectively, in pregnant women from Shandong Province and 0.6 and 11.1%, respectively, in pregnant women from Jiangsu Province, China [54, 55]. In contrast, the positivity rate of anti-HEV IgM was 2.56% in pregnant women in Yunnan Province [56]. These results may reflect the varied seroprevalence of HEV infection in different areas of China. In addition, anti-HEV IgM seropositivity occurs first and persists for 3–5 months after the initial disease onset [57, 58]. Shortly after the appearance of IgM, IgG antibodies develop seropositivity and persist throughout the acute and convalescent phases, maintaining high levels for a year [59]. Therefore, seroprevalence to HEV in pregnant women does not necessarily reflect current HEV-positivity and may result from infection and clearance prior to becoming pregnant. Furthermore, besides the factors related to sex, age structure, and sampling errors, the differences in the specificity and sensitivity of HEV detection attributable to different laboratory diagnostic techniques should also be considered [55, 60]. A recent study among pregnant women in Qinhuangdao, China, showed that the positivity rates of anti-HEV IgM and/or anti-HEV IgG were significantly higher in the third trimester than in the first and second trimesters [61], consistent with previous reports. Among these pregnant women, the HEV strain exclusively identified in those with a history of HEV infection was HEV-4 [61], which seemingly coincides with the fact that HEV-4 is now the most prevalent strain in China. However, considering that HEV-1 and HEV-3 have also been reported in China [62, 63], more studies are needed to clarify the HEV genotypes prevalent among pregnant women in China and the correlation between HEV genotypes and maternal and fetal outcomes.

The combination of other virus infections with HEV infection may enhance the mortality rate. It is estimated that in patients suffering from chronic liver disease (e.g., hepatitis B virus (HBV) infection), these infections often progress to liver failure, with a mortality rate of 27% [64, 65]. Our unpublished data suggested that there were increased rates of other virus infections in both anti-HEV IgM- and anti-HEV IgG-positive pregnant women. In pregnant women with chronic hepatitis C virus (HCV) coinfection, a marked increase in anti-HEV IgG seropositivity and a significant worsening of the biochemical liver indices have been observed [66]. Therefore, more efforts are needed to investigate the epidemiology of other virus infections combined with HEV infection in pregnancy.

### Clinical manifestations

#### Acute hepatitis E

According to previous data, the clinical manifestations of HEV infection are different, varying from asymptomatic to fulminant hepatic failure. HEV usually causes an acute self-limiting hepatitis. The prodromal phase lasts up to 1 week, and the symptoms may be nonspecific, such as malaise, fever, joint pain, nausea, and vomiting. Subsequently, a series of typical symptoms related to acute icteric hepatitis, including jaundice, darkened urine, and pale stools may occur, similar to those seen in HAV infection [67]. However, compared with HAV, patients with HEV are known to have prolonged cholestasis [68]. Mansuy et al. [69] studied 62 confirmed cases of acute hepatitis E over a 5 year period in south-west France and found that about 60% of patients became jaundiced. In fact, the jaundice rate of acute HEV infection is likely much lower given that most cases are not recognized or diagnosed. Serum alanine aminotransferase (ALT) and aspartate aminotransferase (AST) levels are markedly elevated, and bilirubin levels can also rise [10, 70]. In immunocompetent individuals, the infection and symptoms typically resolve spontaneously within 4–6 weeks [71]. Compared to nonpregnant women, clinical features do not differ in pregnant women at first [72]. However, acute liver failure can develop rapidly in patients with HEV infection during pregnancy. Several complications of fulminant hepatic failure, including cerebral edema, DIC, and encephalopathy, appear to occur at a higher rate [18]. Hepatic encephalopathy is the most common cause of death among these patients [72, 73]. Besides maternal mortality, fetal mortality can occur, especially during the third trimester. Preterm delivery, low birth weight, and stillbirth of the fetus or newborn have also been observed in these patients [72].

#### Chronic hepatitis E

Chronic HEV infection is defined as an HEV viremia for more than 3 months [74]. In immunocompromised individuals who receive immunosuppressive therapy following solid organ transplantation or stem cell transplantation [75–77] and those with human immunodeficiency virus (HIV) infection [78, 79] or with hematological malignancy [80–82], chronic HEV infection may develop. This disease course of chronic infection has mainly been reported for HEV-3 and 4, leading to life-threatening liver fibrosis and cirrhosis [75, 80, 83–85]. It has been reported that HEV-3 can cause acute hepatitis E in pregnant women, followed by rapid clearance without any signs of severe courses [50, 51].

#### Extrahepatic manifestations of hepatitis E

Acute or chronic or previous HEV infection can cause extrahepatic manifestations [86, 87], which include a range of neurological symptoms and impaired kidney function associated with cryoglobulinemia [84, 88]. Other extrahepatic manifestations documented in the literature include acute thyroiditis [89], thrombocytopenia [90], and acute pancreatitis [91]. However, it remains uncertain whether extrahepatic manifestations of HEV are attributed to direct infection of extrahepatic tissues or cross-reactive immune responses. As of now, the molecular mechanism of extrahepatic tissue injury caused by HEV infection is unclear, and the roles of pathogenic genotypes and immune function remain to be identified.

### Mechanisms underlying severe liver injury due to HEV infection during pregnancy

**Fig. 2:**
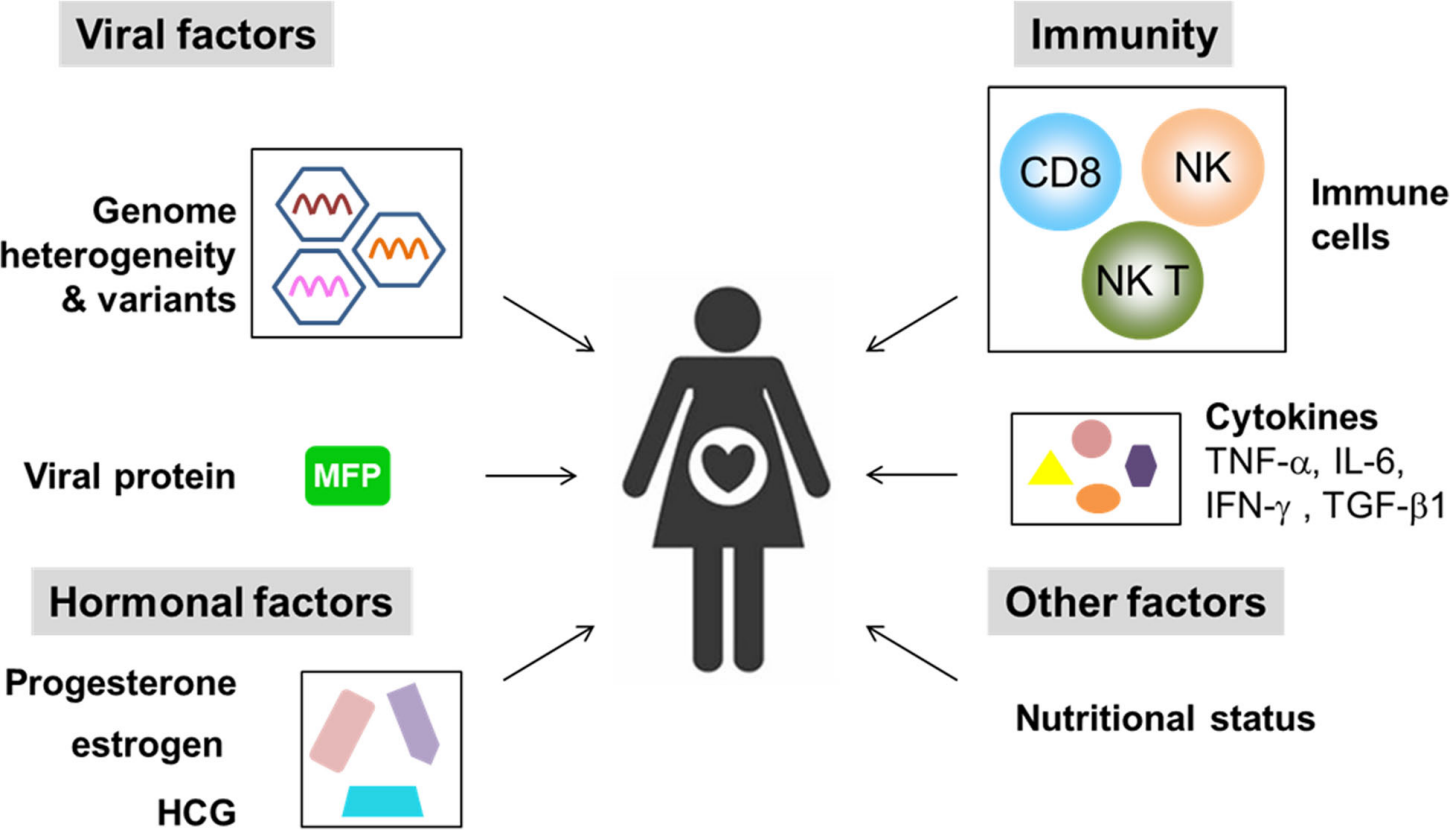
Mechanisms underlying severe liver injury due to HEV infection in pregnancy. The altered status of immunity, hormone levels and viral factors may be related to the severity of the disease. Viral factors include HEV genome heterogeneity and variants, as well as viral proteins, such as MFP, encoded by HEV ORF3, may be associated with the severity of the disease. Host immune factors, such as CD8+ T cells, NK and NKT cells, may be involved in the pathogenicity of HEV during pregnancy. In addition, some cytokines, such as TNF-α, IL-6, IFN-γ, and TGF-β1, may also be involved in this process. The dramatically elevated levels of hormonal factors, including progesterone, estrogen and HCG, in pregnancy may also contribute to liver injury. Other host factors, such as nutritional status, may also influence the severity of HEV infection in pregnant women

The mechanisms of severe liver injury due to HEV infection in pregnant women are unknown. According to previous studies, the unique characteristics of pregnant women, such as altered immunity, hormone levels, and viral factors, such as HEV genome heterogeneity and variants, may be related to the severity of the disease [19, 42] (Fig. 2).

#### Immunity

During pregnancy, the maternal immune system must balance the need to maintain robust immune reactivity to protect both the mother and fetus from invading pathogens and tolerate highly immunogenic paternal alloantigens to sustain fetal integrity [92]. Therefore, the immune alterations that occur during pregnancy are considerably complex. A longitudinal study showed that compared to the postpartum period, the number and activity of natural killer (NK) and T cells decrease during pregnancy, while monocytes, granulocytes, and dendritic cells (DCs) increase in peripheral blood during pregnancy [93], suggesting that adaptive immune responses are weakened, while innate immune responses are strengthened during pregnancy [93–95]. Additionally, a shift from a Th1-dominated immune response to a Th2-dominated response, known as a “Th2 bias”, during pregnancy has been proposed [96]. Th2 cells stimulate B lymphocytes and increase antibody production but inhibit the cytotoxic T lymphocyte response, leading to impaired cell-mediated immunity [96]. In view of these points, the pregnant mother and the fetus may be protected from susceptibility to initial infection. However, once infected, pathogen clearance may be impaired, resulting in increased severity of the disease, especially for infections such as influenza, cytomegalovirus (CMV), severe acute respiratory syndrome (SARS), varicella- zoster, malaria, and herpes simplex virus (HSV) infection, in which cell-mediated immunity is important [93, 97]. Regarding the regulation of cellular immunity during pregnancy, the Th1 / Th2 paradigm has been expanded into the Th1 / Th2 / Th17 and Treg cell paradigm to better explain the mechanism by which the fetus is not rejected by maternal immune cells [98]. The function of effector T cells, such as Th1, Th2, and Th17 cells, is regulated by CD4+ CD25+ Treg cells [99, 100]. Therefore, although the Th1/Th2 paradigm is useful, a host of important immune elements operates during pregnancy to regulate systemic Th1 responses, including higher frequencies of T regulatory cells.

The immunological mechanisms underlying severe liver injury due to HEV infection in pregnancy are not yet well understood. It has been reported that liver injury resulting from HEV in the general population is due to immune-activated CD8, NK and NKT cells in the liver as well as in peripheral circulation [86, 101–103]. In HEV-positive pregnant patients with FHF, CD4 + T cell counts were lower, CD8 + T cell counts were higher and CD4+/CD8+ ratios were significantly lower than those in HEV-negative pregnant women or in controls with FHF [104]. However, the role of NK and NKT cells in the pathogenesis of FHF in pregnant women remains unknown. Moreover, although Th2 bias was confirmed to exist in pregnant women with HEV infection, its effect on the severity of HEV infection is unknown [86].

Impaired monocyte-macrophage functions and defective Toll-like receptor signaling have been found in HEV-infected pregnant women with ALF, suggesting that inadequate triggers for innate immune responses may contribute to the development and severity of liver injury due to HEV infection in pregnancy [86, 105]. In pregnant patients with FHF due to HEV, the DNA-binding activity of NF-κB was much higher than those in nonpregnant women and women with acute viral hepatitis (AVH) without FHF. An analysis of the NF-κB complex showed that the expression of p65 was completely absent or dramatically decreased in these patients [106]. High concentrations of cytokines, including TNF-α, IL-6, IFN-γ, and TGF-β1, may also be related to adverse pregnancy outcomes [107]. Therefore, the abnormal expression or functioning of immunological factors may cause deregulated immunity and severe liver damage in the context of HEV infection. Other host factors, such as nutritional status, including micronutrient deficiencies and the lack of folic acid, may also influence the immune response to HEV infection in pregnant women [108–111].

#### Hormonal factors

As pregnancy progresses, the levels of hormonal factors, such as progesterone, estrogen and human chorionic gonadotropin (HCG), change dramatically and are considerably higher than at any other time [96, 112, 113], potentially also contributing to poor outcomes. The levels of the three hormonal factors were found to be higher in HEV-positive pregnant FHF patients than in HEV-negative patients or controls [104]. Increased estradiol in the serum of HEV-infected pregnant women promoted viral replication [114, 115], which may be associated with poor outcomes [86]. In addition, a high level of estrogen was found to be related to preterm delivery, low birth weight infants and fetal mortality through placental dysfunction in pregnant women with HEV infection [116].

Many studies have shown that hormonal factors play an important role in the modulation of the immune system. For example, high levels of progesterone and estrogen can alter the balance between Th1 and Th2 responses [96]. Estrogen also directly reduces CD8 T cell cytotoxicity [94]. Moreover, estrogen can alter B cell survival and activation as well as suppress B cell lymphopoiesis during pregnancy [94]. Progesterone receptor (PR) gene mutations (PROGINS) were reported to be associated with the incidence of FHF in pregnancy. Progesterone-induced blocking factor (PIBF), which exerts antiabortive activity by inhibiting NK cells and influencing both humoral and cellular immune responses, along with PR was found to be decreased in women with FHF [86, 117].

#### Viral factors

##### Genome heterogeneity and HEV variants

According to the epidemiology of HEV infection, there were variations in the rates of maternal morbidity and mortality among the different HEV genotypes. According to current data, HEV-1 and HEV-2 are the most common genotypes associated with morbidity and mortality in pregnancy. In contrast, the association of increased incidence and severity of hepatitis E in pregnant women infected with HEV-1 or HEV-2 is not observed for HEV-3 and HEV-4 [10, 49–51, 118, 119]. A recent study in an ex vivo model using organ culture from the maternal decidua and fetal placenta demonstrated that HEV-1 replicated more efficiently at the maternal-fetal interface, produced more infectious progeny virions, and caused more severe tissue alterations than HEV-3 [120]. These results indicate that the HEV genotype may play an important role in the pathogenicity of HEV, especially during pregnancy. However, due to the lack of appropriate in vivo and in vitro experimental models and the difficulty of propagating the virus in vitro, no studies have systematically compared the susceptibility, infectivity, replication ability, and virulence of the different HEV genotypes.

In addition, genetic HEV variants have shown to affect viral morphogenesis, pathogenesis, clinical outcomes, and antiviral resistance [121–123]. However, it remains unknown whether these variants have been linked to morbidity/mortality in pregnancy, and needs further study.

##### Viral proteins

The detailed functions of the viral proteins in HEV infection and pathogenesis remain unclear. The D29N and V27A mutations in ORF1-encoded Met were significantly associated with poor outcomes in acute liver failure patients [124]. V239A mutation in ORF1-encoded Hel identified in severe hepatitis cases might be associated with increased virulence of the genotype 3 virus [125]. ORF1-encoded macrodomain can inhibit the phosphorylation/activation of interferon regulatory factor 3 (IRF-3), thus possibly combating the host antiviral response [126]. In addition, the macrodomain can interact with the light chain subunit of human ferritin and inhibit its secretion, suggesting its possible role in suppressing the innate immune response [127]. These reports suggest the possible involvement of ORF1-encoded proteins in determining the outcome of HEV infections. Besides ORF1, the ORF2-encoded capsid protein can also antagonize IFN induction by blocking the phosphorylation of IRF-3 via interaction with the multiprotein complex consisting of mitochondrial antiviral-signaling protein (MAVS), TANK-binding kinase 1 (TBK1), and IRF3 [128]. Recently published data showed that another ORF2-encoded protein, which is glycosylated and can be secreted into the patient serum or supernatants of HEV-infected cell culture in the form of a dimer, inhibits antibody-mediated neutralization and thus may lead to immune escape [35]. These studies revealed that ORF2-encoded proteins might be involved in the interference and/or evasion of the hosts’ innate immunity. In addition, some studies have shown that capsid protein may play a role in facilitating HEV survival or replication in infected hepatocytes. For example, capsid protein inhibits cellular NF-κB activity by blocking ubiquitin-mediated proteasomal degradation of IκBα in human hepatoma cells, presumably enhancing survival of HEV-infected hepatocytes [129]. Moreover, the capsid protein activates the pro-apoptotic gene CHOP and anti-apoptotic heat shock proteins [130]. HEV ORF3 is proposed to be responsible for HEV-associated coagulopathy. ORF3-encoded proteins may interact with several clotting-related pathways, leading to an imbalance in coagulation and fibrinolysis [131].

### Management of HEV infection during pregnancy

Ribavirin was classified in Pregnancy Category X by the United States Food and Drug Administration (FDA) because of its embryocidal and teratogenic effects in animals [25, 132, 133] Thus, ribavirin is not recommended for use in pregnant women. IFN-α was classified in Pregnancy Category C by the FDA, taking into account its abortifacient effect in animals and adverse effects [133]. Therefore, IFN-α was not recommended to be administered to pregnant women [23, 24]. Recently, sofosbuvir showed antiviral activity against HEV both in vitro and in vivo and thus may be a promising antiviral drug against HEV in pregnancy as a pregnancy category B drug [134, 135]. As far as other antiviral candidates are concerned, interferon λ1–3 was shown to inhibit HEV replication [136, 137]. The antisense peptide-conjugated morpholino oligomers (PPMO) HP1, targeting a highly conserved sequence in the start site region of ORF1, can lead to a significant reduction in the levels of HEV RNA and capsid protein, suggesting its potential as a promising antiviral candidate [138]. A recent study showed that nucleoside analogs NITD008, 2′-C-methylguanosine (2CMG), and the non-nucleoside inhibitor GPC-N114 can inhibit HEV in cell culture [139, 140]. However, more controlled studies are needed before sofosbuvir can be recommended for HEV infection in pregnancy. For the other antiviral candidates mentioned above, there is a long way to go. Therefore, the management of HEV infection in pregnancy is currently supported by diligent monitoring and intensive care [42].

Considering that there is no established treatment available for HEV infection in pregnant women, preventing HEV infection in pregnancy may be the most important management strategy [141]. The Chinese vaccine for HEV, HEV 239, has been demonstrated to be protective against both HEV-1 and HEV-4 [122, 142]. In healthy adults, three doses of HEV 239 (30 μg of purified recombinant hepatitis E antigen absorbed to 0.8 mg of aluminium hydroxide suspended in 0.5 mL of buffered saline) were administered intramuscularly at 0, 1, and 6 months and 100.0% vaccine efficacy was achieved [16]. HEV 239 also seems to be safe in pregnant women [143]. In addition, in a rabbit model, two doses of 10 μg or 5 μg HEV 239 vaccine administered intramuscularly on weeks 0 and 4 not only serve to protect pregnant rabbits from HEV infection but also prevent HEV-related adverse outcomes [144]. Although the safety and efficacy of this vaccine in a large population of pregnant women remain to be determined, this vaccine is promising for HEV infection prevention in pregnancy and may thus decrease HEV-associated morbidity and mortality. Besides advocating for studies of HEV 239 vaccination during pregnancy, it would also seem prudent to advocate for vaccination of at-risk women of childbearing age in endemic regions before they become pregnant. Recently, a phase IV trial has been initiated to assess the effectiveness, safety, and immunogenicity of the HEV 239 vaccine in women of childbearing age in rural Bangladesh, where HEV infection is endemic [145].

## Conclusions

HEV infection is not limited to certain developing countries; it is also endemic in many high-income countries and is largely zoonotic in nature. Despite increasing knowledge, many questions regarding HEV, especially HEV infection during pregnancy, remain unanswered. In addition to immune and hormonal factors, genome heterogeneity and HEV variants may be related to the severity of HEV infection in pregnancy. Therefore, it may be important to investigate the prevalent HEV genotypes and their virulence as well as morbidity in pregnancy; this information could be used to develop guidelines for the intervention and management of HEV infection in pregnancy. HEV can be transmitted via blood transfusion. Increasing prevalence rates of HEV in voluntary blood donors (VBDs) and transfusion-associated HEV infections have been reported [1, 4, 10, 146]. Therefore, screening donors for HEV RNA may need to be considered.

Considering that HEV infection in pregnant women may progress to fulminant hepatitis, especially in the third trimester, with a high mortality rate, screening for and monitoring HEV infection early in pregnancy should become a focus. In addition, it is also necessary to inform pregnant women of the potential effects of HEV on the fetus; they should be advised to avoid eating and drinking contaminated food and water to prevent possible HEV exposure. An HEV vaccine may hold great promise for reducing HEV-associated mortality in pregnant women. Finally, there is also a substantial need for novel therapies to treat HEV in pregnancy.

## Data Availability

Not applicable.

## Abbreviations

HEV: Hepatitis E virus
HEV-1: Genotypes 1
HEV-2: Genotypes 2
HEV-3: Genotypes 3
HEV-4: Genotypes 4
IFN-α: Interferon-α
FHF: Fulminant hepatitis failure
eHEV: Enveloped HEV
UTR: Untranslated region
ORFs: Open reading frames
Met: Methyltransferase
Y: Y-domain
PCP: Papain-like cysteine protease
HVR: Hypervariable region
X: Macrodomain
Hel: RNA helicase
RdRp: RNA-dependent RNA polymerase
DIC: Disseminated intravascular coagulation
HBV: Hepatitis B virus
HCV: Hepatitis C virus
ALT: Alanine aminotransferase
AST: Aspartate aminotransferase
HIV: Human immunodeficiency virus
NK: Natural killer
DCs: Dendritic cells
CMV: Cytomegalovirus
SARS: Severe acute respiratory syndrome
HSV: Herpes simplex virus
AVH: Acute viral hepatitis
HCG: Human chorionic gonadotropin
PR: Progesterone receptor
PROGINS: Progesterone receptor (PR) gene mutations
PIBF: Progesterone-induced blocking factor
IRF-3: Interferon regulatory factor 3
MAVS: Mitochondrial antiviral-signaling protein
TBK1: TANK-binding kinase 1
FDA: Food and Drug Administration
PPMO: Peptide-conjugated morpholino oligomers
2CMG: 2′-C-methylguanosine
VBDs: Voluntary blood donors
